# EndoVAC Therapy for Post-TEVAR Secondary Esophageal Fistula: A Rare Case of Delayed Secondary Esophageal Fistula After TEVAR Managed with Endoluminal Vacuum Therapy

**DOI:** 10.3390/life16030460

**Published:** 2026-03-11

**Authors:** Bogdan-Mihnea Ciuntu, Andreea Ludușanu, Adelina Tanevscki, Rareș Ștefan Costârnache, Mihaela Corlade-Andrei, Petru Radu Soroceanu, Dan Vintilă, Irina Mihaela Abdulan, Mihai-Lucian Zabara, Gheorghe Balan

**Affiliations:** 1 Department of General Surgery, Faculty of Medicine, University of Medicine and Pharmacy “Grigore T. Popa”, 700115 Iasi, Romania; bogdan-mihnea.ciuntu@umfiasi.ro (B.-M.C.); andreealudusanu1106@yahoo.com (A.L.); papancea.adelina@umfiasi.ro (A.T.); petru.soroceanu@umfiasi.ro (P.R.S.); dan.vintila@umfiasi.ro (D.V.); mihai-lucian.zabara@umfiasi.ro (M.-L.Z.); 2 General Surgery Clinic, “St. Spiridon” County Emergency Clinical Hospital, 1 Independence Boulevard, 700111 Iasi, Romania; 3 Faculty of Medicine, University of Medicine and Pharmacy “Grigore T. Popa”, 700115 Iasi, Romania; 4 Department of Emergency Medicine, Surgery II, University of Medicine and Pharmacy “Grigore T. Popa”, 700115 Iași, Romania; mihaela.corlade2@umfiasi.ro; 5 Department of Medical Specialties I, University of Medicine and Pharmacy “Grigore T. Popa”, 700115 Iasi, Romania; 6 Department of Gastroenterology, University of Medicine and Pharmacy “Grigore T. Popa”, 700115 Iași, Romania; gheorghe-g-balan@umfiasi.ro

**Keywords:** aorto-esophageal fistula, secondary esophageal fistula, thoracic endovascular aortic repair (TEVAR), endograft erosion, multidisciplinary approach

## Abstract

**Background:** Aorto-esophageal fistula is a rare but life-threatening condition most often linked to thoracic aortic aneurysms and significant upper gastrointestinal bleeding. Thoracic endovascular aortic repair (TEVAR) is a crucial, life-saving procedure, but delayed complications, such as secondary esophageal fistulas caused by endograft erosion, can develop. Prompt recognition and multidisciplinary management are vital for survival. **Case Presentation:** We describe a 57-year-old patient with cardiovascular comorbidities and a saccular thoracic aortic aneurysm, who initially presented with massive hematemesis, melena, and hemodynamic instability. Imaging showed an aorto-esophageal fistula. Emergency treatment included placing a fully covered esophageal stent followed by TEVAR. Three weeks later, he experienced fever, chest pain, and worsening dysphagia. Laboratory tests indicated elevated inflammatory markers and hypoalbuminemia. Computed tomography revealed a new retrocardial esophageal fistula at T9, caused by mechanical erosion from the thoracic endograft. Endoluminal vacuum-assisted closure (EndoVAC) therapy was performed, leading to clinical improvement and the return of esophageal function. **Conclusions:** This case highlights a rare instance of a delayed secondary esophageal fistula after TEVAR beneath a preexisting stent, likely due to chronic contact between the endograft and esophagus. Despite advancements in endoscopic therapy, secondary fistulas after TEVAR are associated with high morbidity. Early diagnosis, aggressive infection management, structured nutritional support, and a multidisciplinary approach are essential. Extraluminal or intraluminal vacuum-assisted closure offers a promising minimally invasive option for managing complex esophageal defects.

## 1. Introduction

Aorto-esophageal fistula (AEF) is a rare but life-threatening condition, most often associated with thoracic aortic aneurysms, penetrating aortic ulcers, or complications following aortic surgery. Although it is infrequent, it is one of the deadliest causes of upper gastrointestinal bleeding, with mortality rates over 80% in cases of delayed diagnosis or treatment [1]. Patients typically present with the classic triad of chest pain, sentinel hemorrhage, and massive hematemesis, although symptoms can vary and may be nonspecific, making early detection difficult. Therefore, early diagnosis and prompt intervention are essential for improving survival chances [2].

Thoracic endovascular aortic repair (TEVAR) has become a preferred treatment option for hemodynamically unstable patients, providing quick aneurysm exclusion, hemorrhage control, and reduced perioperative morbidity compared to open surgical repair [3]. Despite these benefits, TEVAR carries the risk of both early and late complications, such as endoleaks, graft migration, infection, and, more rarely, secondary esophageal injury. Secondary esophageal fistula after TEVAR—a complication different from primary AEF—is extremely rare and linked with high morbidity and mortality [4,5]. The underlying mechanisms include chronic mechanical compression, direct erosion from the endograft, ischemia of the esophageal wall, mediastinal infection, and postoperative inflammatory changes [5].

The clinical signs of secondary esophageal fistulas are often subtle and nonspecific, including fever, chest discomfort, and dysphagia to signs of systemic infection and sepsis [6]. Delayed recognition can lead to rapid clinical deterioration, massive bleeding, or mediastinitis. Imaging, especially contrast-enhanced computed tomography, is essential for early detection and helps identify the fistula tract, associated mediastinal collections, and possible endograft involvement [6,7]. Managing these cases is complex and requires personalized strategies that combine endoscopic procedures, surgical approaches, infection control, and critical care support [7]. Traditional treatments such as open surgical repair or esophageal exclusion have high morbidity, especially in patients with significant comorbidities, emphasizing the importance of minimally invasive options [8].

Given the rarity and clinical severity of secondary esophageal fistulas after TEVAR, each reported case offers valuable insights into their diagnosis, management, and outcomes [9,10]. Here, we present a complex case of delayed esophageal fistula occurring three weeks after emergency TEVAR for a primary aorto-esophageal fistula. The case highlights the intricate pathophysiological processes, diagnostic challenges due to overlapping clinical features, and a multidisciplinary management approach, including the use of endoluminal vacuum-assisted closure (EndoVAC) therapy as a minimally invasive salvage technique.

## 2. Case Report

We present the case of a 57-year-old male with a history of arterial hypertension and a saccular thoracic aortic aneurysm with intraluminal thrombus. The patient was admitted to the emergency department with massive hematemesis, melena, severe retrosternal and epigastric pain radiating to the neck, and profound fatigue. Upon presentation, he appeared pale, dehydrated, and hemodynamically unstable. Laboratory tests showed severe anemia, significantly elevated C-reactive protein, and signs of systemic inflammation.

To identify the source of bleeding and evaluate thoracic involvement, contrast-enhanced thoracic and abdominal CT scans were performed. Imaging showed contrast extravasation from the saccular aneurysm of the descending thoracic aorta directly into the esophageal lumen, consistent with a primary aorto-esophageal fistula. A large amount of blood was present in the stomach.

**Figure 1:**
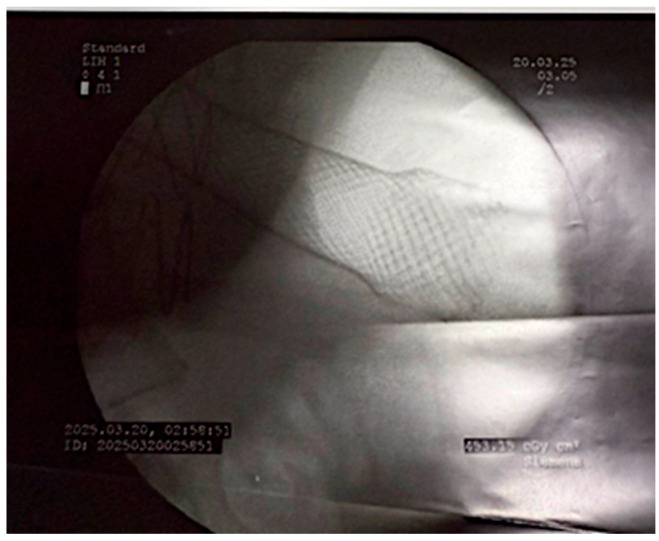
Radiological visualization of the fully covered esophageal stent.

Following hemodynamic stabilization and initial hemorrhage control, a fully covered self-expandable metal stent (SEMS) was endoscopically deployed to seal the aorto-esophageal communication and protect the esophagus from further contact with blood and aneurysmal contents (Figure 1). Under general anesthesia and fluoroscopic guidance, flexible esophagoscopy was first performed to locate the lesion. A guidewire was advanced across the defect, and the stent was delivered over it. The stent was sized to fully cover the fistulous area, extending several centimeters proximally and distally to ensure complete coverage while avoiding migration. Care was taken to position the proximal end below the upper esophageal sphincter and the distal end above the gastroesophageal junction. Post-deployment, stent position was confirmed under fluoroscopy, and a water-soluble contrast swallow verified fistula exclusion and the absence of leakage. Enteral access was maintained via a nasogastric tube, and oral intake was initially withheld to facilitate mucosal healing.

After placing the fully covered esophageal stent via endoscopy, the patient was instructed to remain nil per os for 14 days to reduce mechanical stress on the esophageal wall and promote optimal healing of the fistulous defect. Parenteral nutrition was administered during this period, and the patient was closely monitored for infection, bleeding, or stent migration. Subsequently, he was transferred to the cardiovascular surgery department to undergo thoracic endovascular aortic repair. An aortic endograft deployment extended from the ascending aorta to the upper abdominal aorta. Pre-procedural planning relied on high-resolution contrast-enhanced computed tomography angiography (CTA), enabling precise assessment of the aneurysm, landing zones, and access vessel anatomy, in line with current expert consensus recommendations [11].

**Figure 2:**
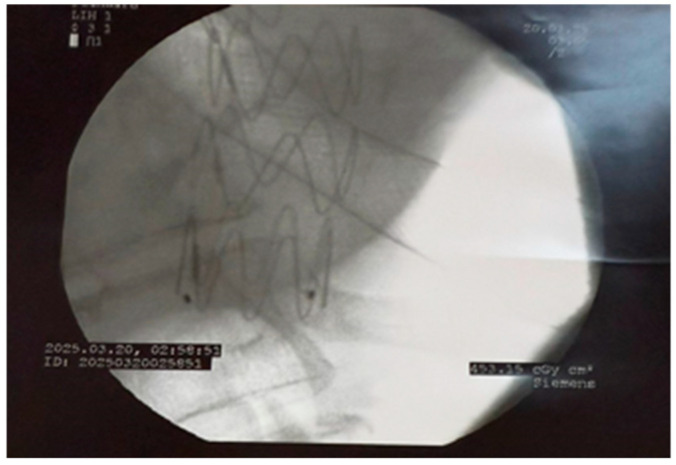
Radiological visualization of the thoracic aortic endograft placed during TEVAR, with the fully covered esophageal stent visible posteriorly, illustrating the spatial relationship between the two devices.

**Figure 3:**
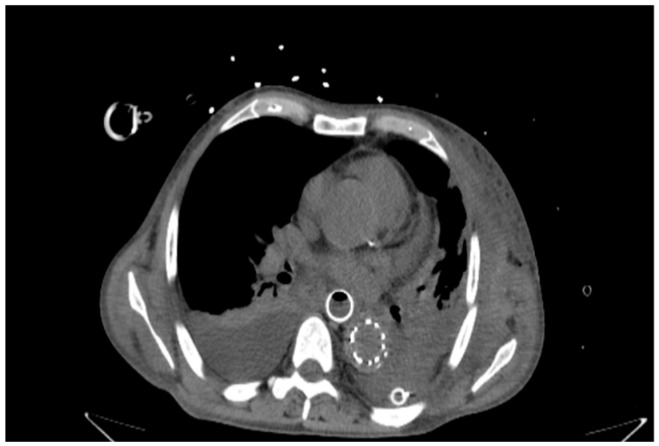
CT scan showing the postoperative setup after emergency treatment. The image displays the fully covered esophageal stent in place and the thoracic aortic endograft.

Vascular access was gained through the common femoral artery, and a stiff guidewire was advanced under fluoroscopic guidance for precise device placement. During deployment, systemic anticoagulation and strict blood pressure control were maintained to reduce the risk of graft displacement [11,12]. The endograft was deployed with slight oversizing (10–15% relative to the reference aortic diameter), as recommended in current guidelines [11,13]. Completion angiography confirmed proper positioning and the absence of endoleaks, and balloon molding was used when necessary to improve graft fit (Figure 2 and Figure 3).

The patient’s immediate postoperative course was favorable, with stable hemodynamics, cessation of acute bleeding, and overall clinical improvement. He was discharged with instructions for close follow-up and scheduled for clinical and imaging assessments at one month to monitor stent position, vascular integrity, and recovery.

Three weeks after the initial hospitalization and successful TEVAR, the patient was readmitted with fever, night sweats, worsening dysphagia, chest pain, and increasing fatigue. On examination, he looked pale, dehydrated, and showed signs of hypokinetic and hypotrophic muscular system, indicating the severity of his systemic decline. Laboratory tests revealed recurrent severe anemia, persistent elevation of C-reactive protein, and hypoalbuminemia.

**Figure 4:**
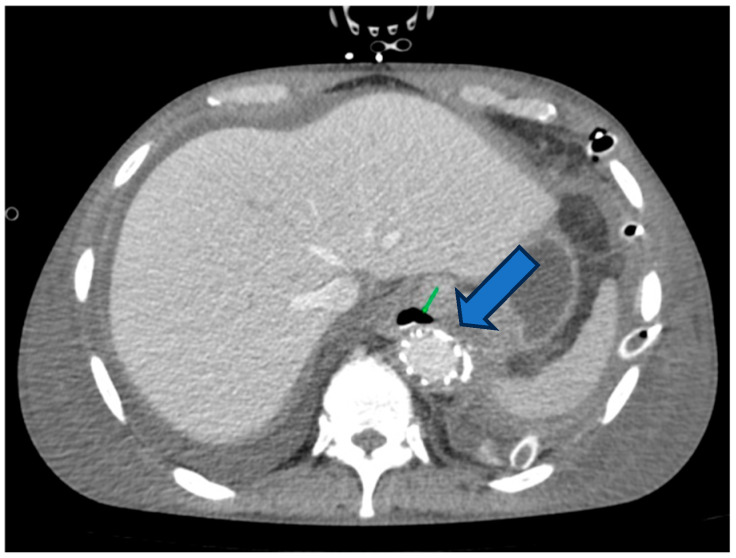
CT scan performed three weeks later, revealing the secondary esophageal fistula–endograft contact point.

Urgent contrast-enhanced thoracic CT revealed a new esophageal defect in the lower third of the esophagus at the T9 vertebral level, positioned retrocardiacally where it closely contacts the thoracic endograft (Figure 4). Oral contrast leak into the mediastinum was observed, along with small air bubbles around the stent-graft, strongly indicating a secondary esophageal fistula caused by mechanical erosion from the endograft. The previously placed fully covered esophageal stent remained in place, while the new defect measured approximately 10 mm in diameter beneath the stent.

Given the high risk of direct surgical repair in a contaminated mediastinal area and the patient’s cardiovascular comorbidities, the multidisciplinary team chose endoscopic management.

The team decided to remove the esophageal stent, assess the defect and cavity, perform lavage and aspiration, and extract necrotic debris. They evaluated the remaining cavity to prepare for the next step.

**Figure 5:**
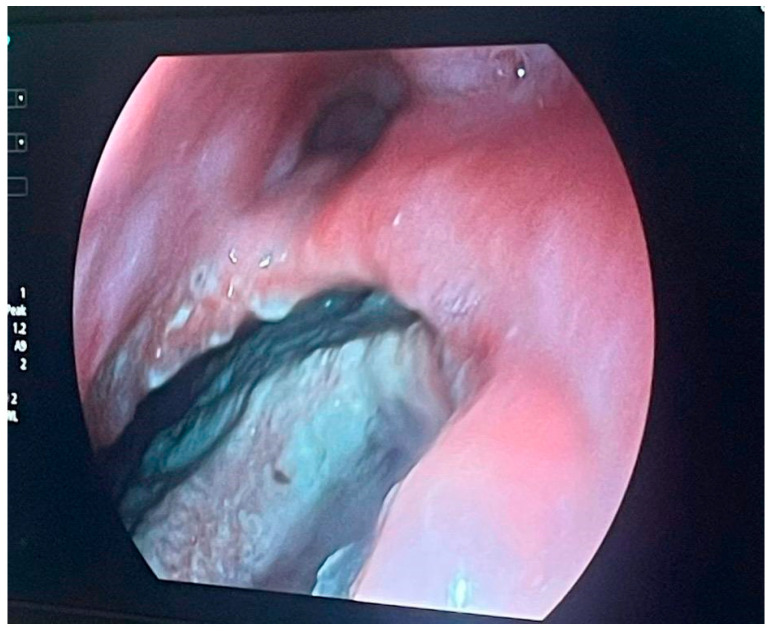
Endoscopic visualization of the 10 mm esophageal defect.

**Figure 6:**
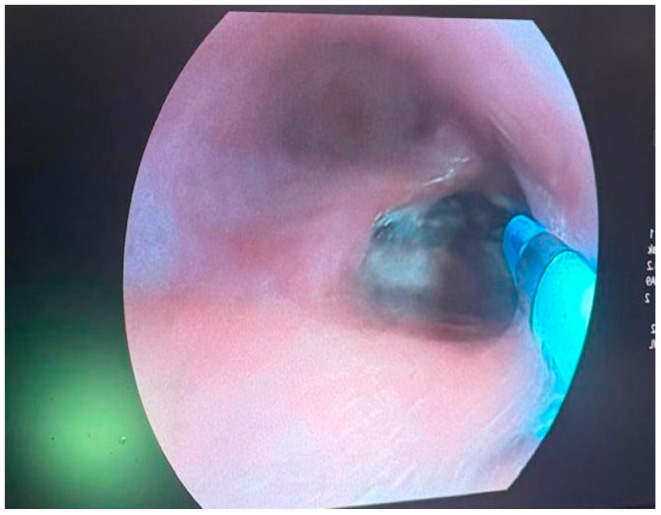
Endoscopic appearance of the esophageal fistula after placement of the EndoVAC sponge.

An endoluminal vacuum-assisted closure system was placed to facilitate continuous drainage, reduce contamination, and encourage granulation tissue formation [14,15]. During the procedure, endoscopic visualization confirmed a round, well-defined esophageal wall defect with direct exposure to the mediastinal cavity, consistent with prior CT findings. Intraoperative photographs documented the fistula margins, its retrocardiac extension, and the placement of the EndoVAC sponge within the defect (Figure 5 and Figure 6).

During the same intervention, when EndoVAC was placed (endoluminal vacuum kit set at −150 mmHg), a feeding jejunostomy was performed, and the patient was subsequently fed enterally through it. The kit was changed every 5 days under general anesthesia. The patient was intubated only during the procedures; otherwise, oxygen was provided via high-flow nasal cannula.

During the EndoVAC procedure, purulent secretions were collected from the fistulous cavity for microbiological analysis. Cultures showed a predominance of multidrug-resistant *Klebsiella pneumoniae*, along with variable growth of *Streptococcus mitis*, *Pseudomonas aeruginosa*, and *Enterococcus* species, indicating a polymicrobial mediastinal infection. Based on these findings, targeted antimicrobial therapy with colistin was started, reflecting both the severity of the infection and the challenges of managing secondary esophageal fistulas after TEVAR.

The cavity was debrided, removing necrotic debris, and reductions in volume along with the appearance of granulation tissue were observed. On day 22, bronchoscopic excision was performed (the patient presented with a respiratory impairment—muco-purulent cough, with the installation of a drain tube). Maximum therapy was continued, with a slow but favorable evolution, and complete closure of the defect was achieved by day 41. The patient was discharged with feeding via jejunostomy and instructions for follow-up at 1 month, 3 months, and 6 months.

The case management was challenging due to a high-risk mediastinal condition in a severely ill patient (esophageal perforation with mediastinal abscess) and notable vascular disease (TEVAR—with a high risk of sepsis).

The technical challenge involved placing the EndoVAC through the esophageal fistula opening. Caution was required because it was very close to the previously positioned stent.

Jejunostomy feeding was necessary to rest the upper digestive tract and also to maintain the patient’s nutritional status.

## 3. Discussion

This case highlights the complexity of secondary esophageal fistula following thoracic endovascular aortic repair, caused by the interaction of mechanical erosion, graft infection, and mediastinal contamination. In our patient, an esophageal defect appeared three weeks after successful TEVAR and placement of a covered esophageal stent, likely due to chronic contact between the endograft and the esophageal wall. This delayed presentation is consistent with reports showing that secondary esophageal fistulas can develop weeks to years after TEVAR, depending on individual factors [15,16].

The pathogenesis is multifactorial: continuous friction or pulsatile stress from the endograft can gradually erode adjacent esophageal tissue, while prosthetic infection weakens surrounding structures. Polymicrobial colonization, particularly by multidrug-resistant organisms, exacerbates tissue destruction, promotes adherence of the graft to mediastinal tissues, and increases the risk of fistulization [17]. In our case, persistent *Klebsiella pneumoniae*, *Enterococcus*, and *Streptococcus* species isolated from the fistula strongly support this infectious-erosive paradigm.

Treatment options for secondary esophageal fistulas after TEVAR remain limited and high-risk. Open surgical procedures—such as graft removal, debridement, and in situ reconstruction—have high perioperative mortality rates, often exceeding 70% in reported series [17,18]. Minimally invasive methods, including endoluminal vacuum-assisted closure, are becoming valuable options due to continuous drainage, reduction in bacterial load, and promotion of granulation tissue while avoiding the morbidity associated with open surgery [18]. Clinical reports show high technical success rates, especially when treatment is started early. For instance, a 76-year-old patient with an esophageal–mediastinal fistula after TEVAR achieved closure within 11 days using EndoVAC combined with nutritional support [19].

Biomechanical factors also contribute to fistula formation. Computational fluid–structure interaction (FSI) models show that rigid aortic endografts change native hemodynamics and vessel-wall mechanics, leading to abnormal contact pressures and shear forces at the graft–esophagus interface [20]. High curvature or angulated segments experience localized stress, and repetitive motion from cardiac pulsatility, respiration, and esophageal peristalsis worsens tissue microtrauma. These insights explain why fistulas can develop even with correct device sizing and placement, independent of obvious technical failure [21,22].

This case emphasizes the importance of a multidisciplinary approach involving vascular surgeons, gastroenterologists, infectious disease specialists, radiologists, and critical care teams. Serial endoscopic assessments, microbiological monitoring, and scheduled EndoVAC sponge changes were crucial for adapting therapy to changing clinical conditions [23,24].

## 4. Conclusions

Secondary esophageal fistulas following thoracic endovascular aortic repair are rare but potentially fatal complications caused by the interaction of mechanical stress, prosthetic infection, and tissue vulnerability. Early detection and prompt intervention are essential to stabilize the patient and prevent catastrophic bleeding. Minimally invasive approaches, such as endoscopic stenting and endoluminal vacuum-assisted closure, provide effective treatments, especially for patients who are poor candidates for open surgery. However, clinical outcomes are heavily affected by infection control, comorbidities, and the extent of local tissue damage.

A multidisciplinary approach—integrating vascular and general surgery, gastroenterology, infectious disease, and critical care—is crucial for optimal patient management. Despite advances in endovascular and endoscopic techniques, mortality rates remain high, emphasizing the need for further research on improved graft designs, infection prevention, and optimized minimally invasive therapies for this severe complication.

## Data Availability

The original contributions presented in this study are included in the article. Further inquiries can be directed to the corresponding author.
